# Supplementary material for: Computational exploration of novel ROCK2 inhibitors for cardiovascular disease management; insights from high-throughput virtual screening, molecular docking, DFT and MD simulation
Source: PLoS One. 2023 Nov 16;18(11):e0294511. doi: 10.1371/journal.pone.0294511 (PMC10653426; doi:10.1371/journal.pone.0294511)
Supplement: S1 File — (DOCX) [file pone.0294511.s001.docx]

**Computational exploration of novel ROCK2 inhibitors for cardiovascular disease management; insights from high-throughput virtual screening, molecular docking, DFT and MD simulation**

Iqra Ali^1,2*^, Muhammad Nasir Iqbal^2^, Muhammad Ibrahim^3^, Ihtisham Ul Haq^4,5,6^, Wadi B Alonazi^7^, Abdul Rauf Siddiqi^1*^

*^1^Department of Biosciences, COMSATS University Islamabad, Islamabad Campus, 45550 Islamabad, Pakistan (*[*iqraali857@gmail.com*](mailto:iqraali857@gmail.com)*)*

*^2^Department of Bioinformatics, The Islamia University of Bahawalpur, 63100 Bahawalpur, Pakistan (*[*nasir.iqbal@iub.edu.pk*](mailto:nasir.iqbal@iub.edu.pk)*)*

*^3^Department of Biosciences, COMSATS University Islamabad, Sahiwal Campus, Pakistan (*[*ibrahim@cuisahiwal.edu.pk*](mailto:ibrahim@cuisahiwal.edu.pk)*)*

*^4^Department of Physical Chemistry and Technology of Polymers, Silesian University of Technology, M. Strzody 9, 44-100 Gliwice, Poland (*[*ihaq@polsl.pl*](mailto:ihaq@polsl.pl)*)*

*^5^Joint Doctoral School, Silesian University of Technology, Akademicka 2A, 44-100 Gliwice, Poland.*

*^6^Programa de Pós-graduação em Inovação Tecnológica, Universidade Federal de Minas Gerais, Belo Horizonte, 30150-240, MG, Brazil.*

*^7^Health Administration Department, College of Business Administration, King Saud University, PO Box 71115, Riyadh, 11587, Saudi Arabia (*[*waalonazi@ksu.edu.sa*](mailto:waalonazi@ksu.edu.sa)*)*

***Corresponding Author**

Abdul Rauf Siddiqi, Iqra Ali

[araufsiddiqi@comsats.edu.pk](mailto:araufsiddiqi@comsats.edu.pk) AND [*iqraali857@gmail.com*](mailto:iqraali857@gmail.com)

**List of Figures**

[S1 Fig. Target Protein evaluation from SAVES Server depicts overall quality factor and correctly and incorrectly folded regions. 3](#_Toc149645752)

[S2 Fig. Domain Architecture of ROCK (ROCK1 and ROCK2 comparison) protein. Domains represented with different colors along number of residues and percent identity. 4](#_Toc149645753)

[S3 Fig. Subcellular localization by DeepLoc-1.0 (upper panel), HSLPred (middle panel) and ESLpred (lower panel) 4](#_Toc149645754)

[S4 Fig. Aligned compounds used to build pharmacophore model. 5](#_Toc149645755)

[S5 Fig: Standard drugs i.e., Fasudil, Y27632, ripasudil and cocrystal compound 3SG make interactions in the binding site of ROCK2. 5](#_Toc149645756)

[S6 Fig. (A) Surface representation of A1 compound (brick red color) in the receptor cavity which represented with forest green color along 2D depiction and superimposition of reference ligand in cyan color (B) showing surface mapping of A2 (orange color) in the binding site of receptor along 2D diagram and superimposed view of reference compound (cyan color). 6](#_Toc149645757)

[S7 Fig. ROC curve for docking validation. Values range from 0 to 1 and higher values close to 1 considered good. Here magenta line represented area under the curve value while black horizontal line depicts 0.5 value which signifies random classification. 7](#_Toc149645758)

[S8 Fig. Protein secondary structure elements (SSE) i.e., alpha-helices marked by orange and the beta-strands were described by cyan. (A) Protein secondary structure elements of A1 system (B) Protein secondary structure elements of A2 system during MD simulation. 8](#_Toc149645759)

[S9 Fig. A timeline representation of interactions and different contacts such as H-bonds, Ionic, Hydrophobic, and Water bridges with A1 (A) and A2 (B). 9](#_Toc149645760)

[S10 Fig. (A)Torsion plot of A1 (B) A2 represent conformational evolution of ligand’s rotatable bonds throughout the simulation. The top panel represents 2d schematic of both A1 and A2 while bottom panel shows dial plot and bar plots. The values of the potential are on the left Y-axis of the chart represented in kcal/mol. 10](#_Toc149645761)

**List of Tables**

[S1 Table. Physiochemical properties of ROCK2 receptor 9](#_Toc148131585)

[S2 Table. Binding site residues of target protein identified via CASTp and MOE's site finder tool. 10](#_Toc148131586)

[S3 Table. Active compounds along their physiochemical properties used to generate pharmacophore query. 10](#_Toc148131587)


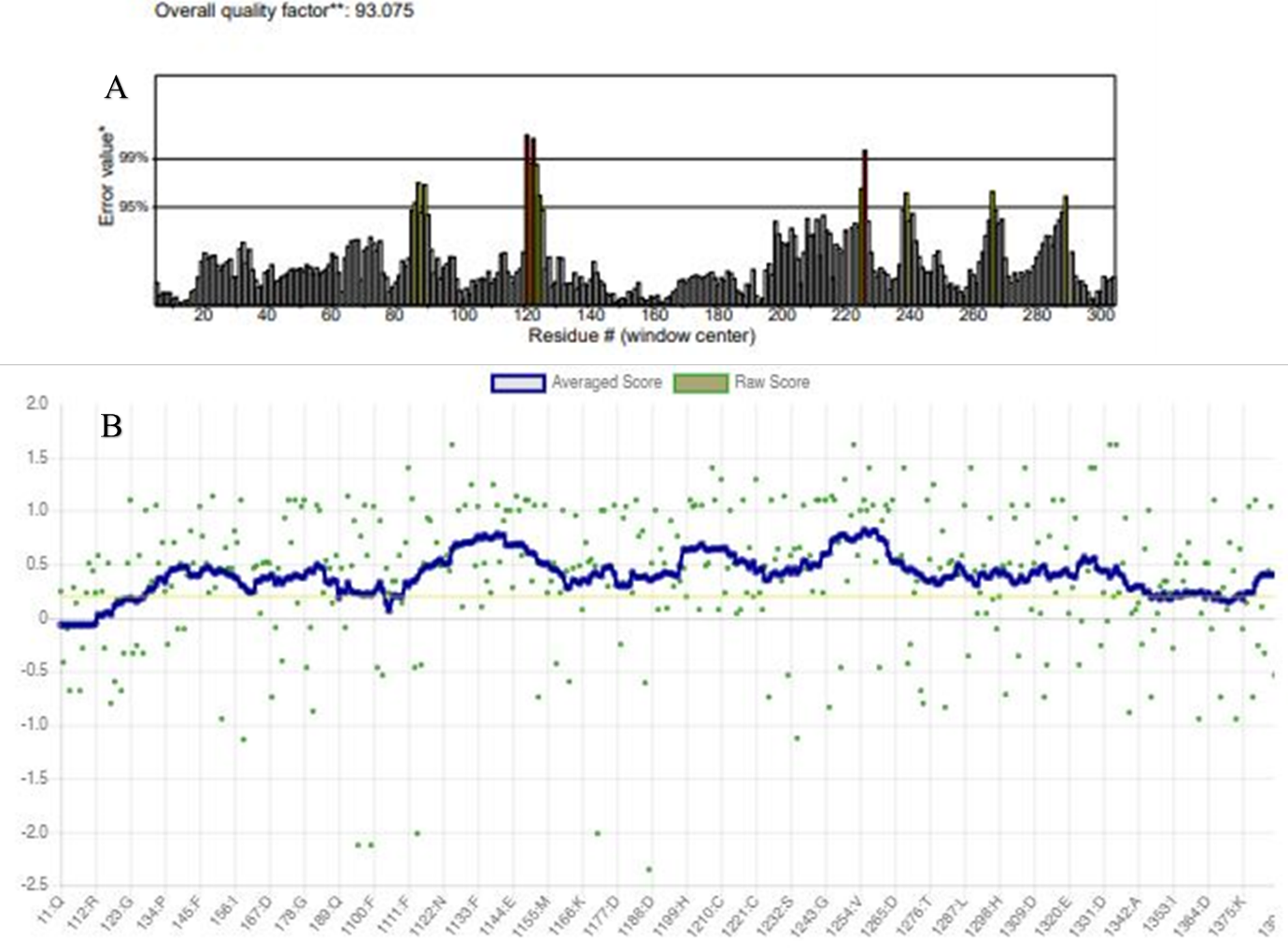


***S1 Fig. Target Protein evaluation from SAVES Server depicts overall quality factor and correctly and incorrectly folded regions***.


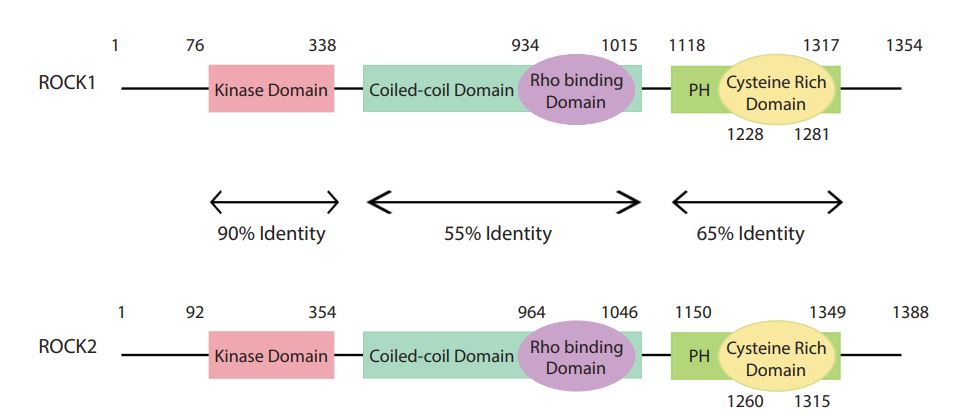


***S2 Fig. Domain Architecture of ROCK (ROCK1 and ROCK2 comparison) protein. Domains represented with different colors along number of residues and percent identity.***


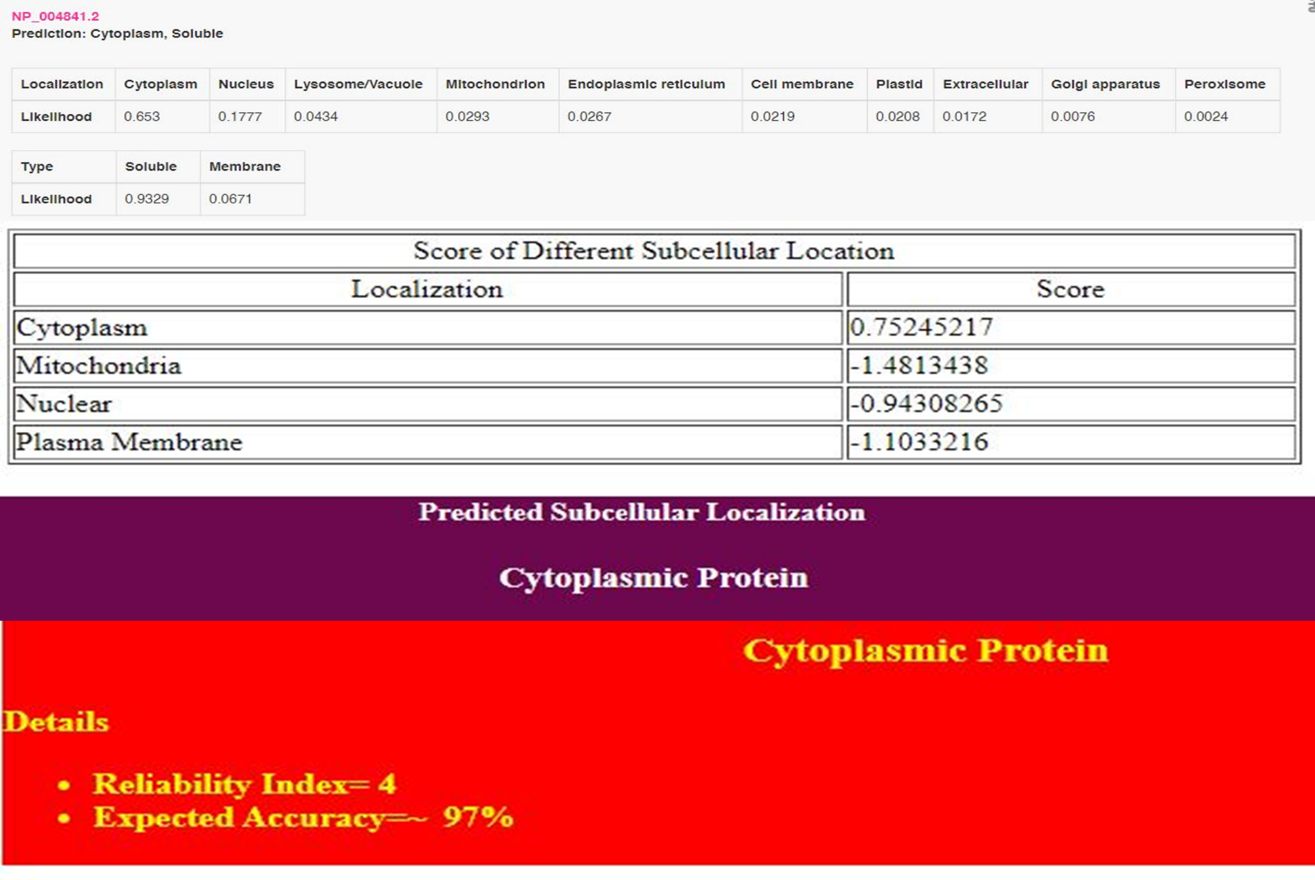


***S3 Fig. Subcellular localization by DeepLoc-1.0 (upper panel), HSLPred (middle panel) and ESLpred (lower panel)***


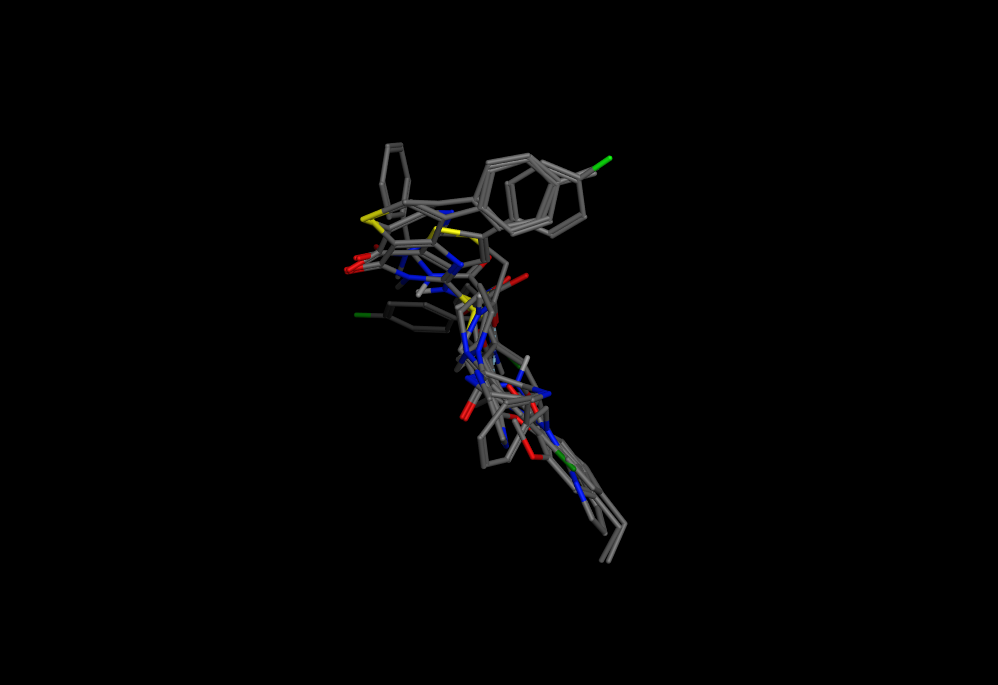


***S4 Fig. Aligned compounds used to build pharmacophore model.***


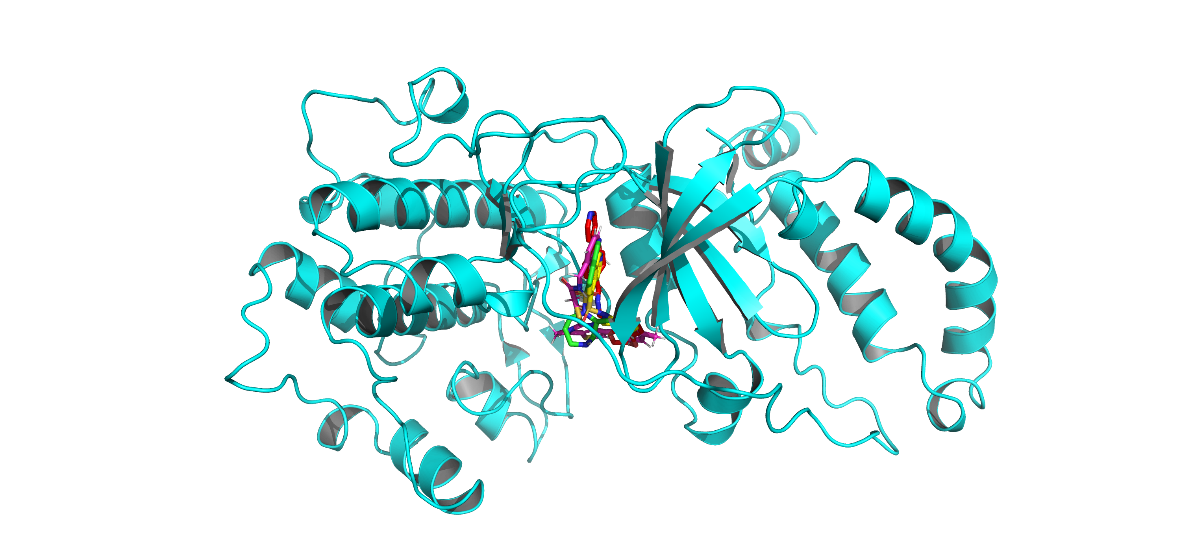


***S5 Fig: Standard drugs i.e., Fasudil (magenta), Y27632 (yellow), ripasudil (green) and cocrystal compound 3SG (red color) make interactions in the binding site of ROCK2.***


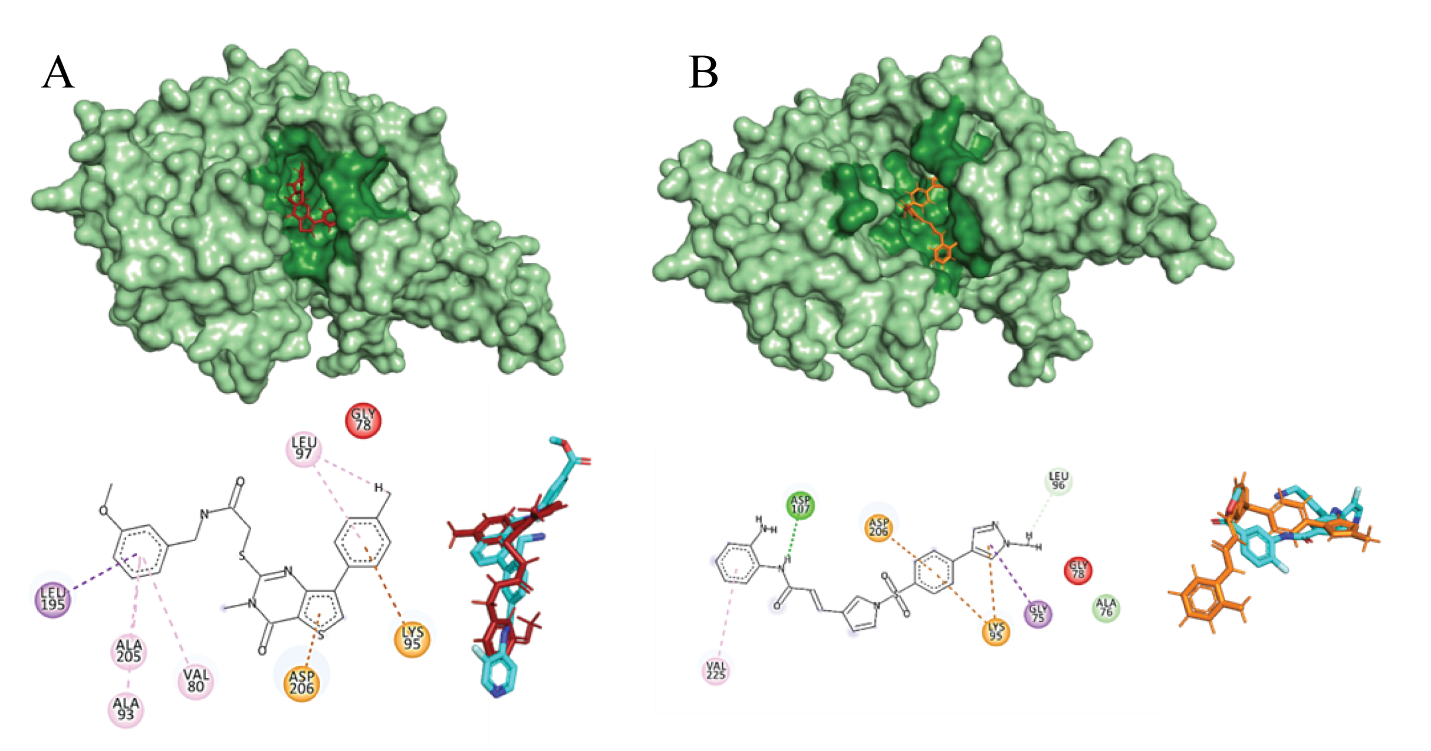


***S6 Fig. (A) Surface representation of A1 compound (brick red color) in the receptor cavity which represented with forest green color along 2D depiction and superimposition of reference ligand in cyan color (B) showing surface mapping of A2 (orange color) in the binding site of receptor along 2D diagram and superimposed view of reference compound (cyan color).***


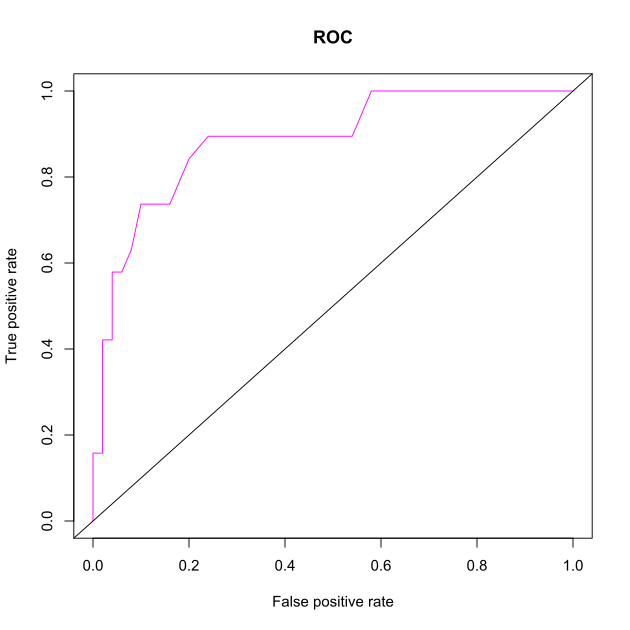


***S7 Fig. ROC curve for docking validation. Values range from 0 to 1 and higher values close to 1 considered good. Here magenta line represented area under the curve value while black horizontal line depicts 0.5 value which signifies random classification.***


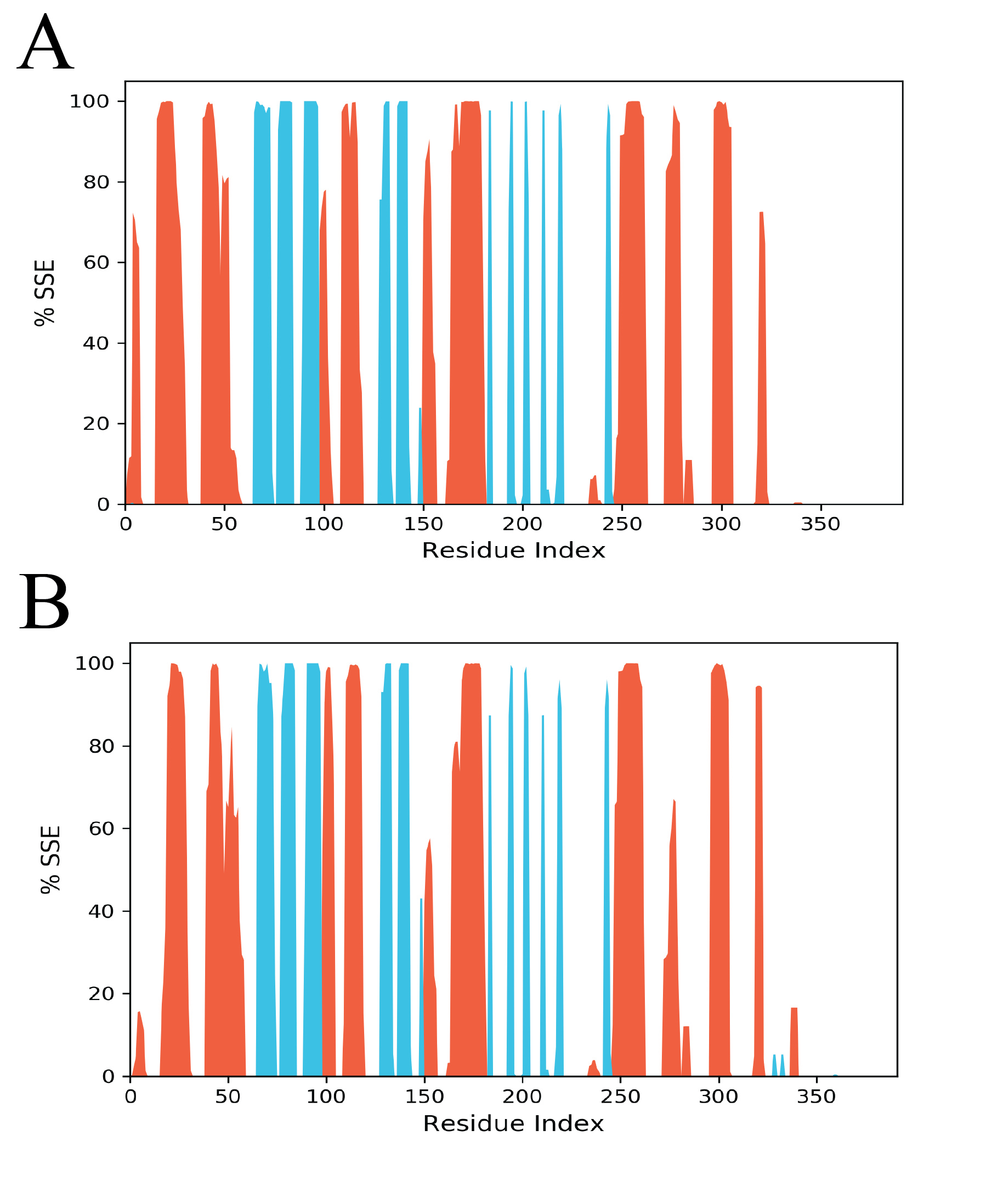


***S8 Fig. Protein secondary structure elements (SSE) i.e., alpha-helices marked by orange and the beta-strands were described by cyan. (A) Protein secondary structure elements of A1 system (B) Protein secondary structure elements of A2 system during MD simulation.***


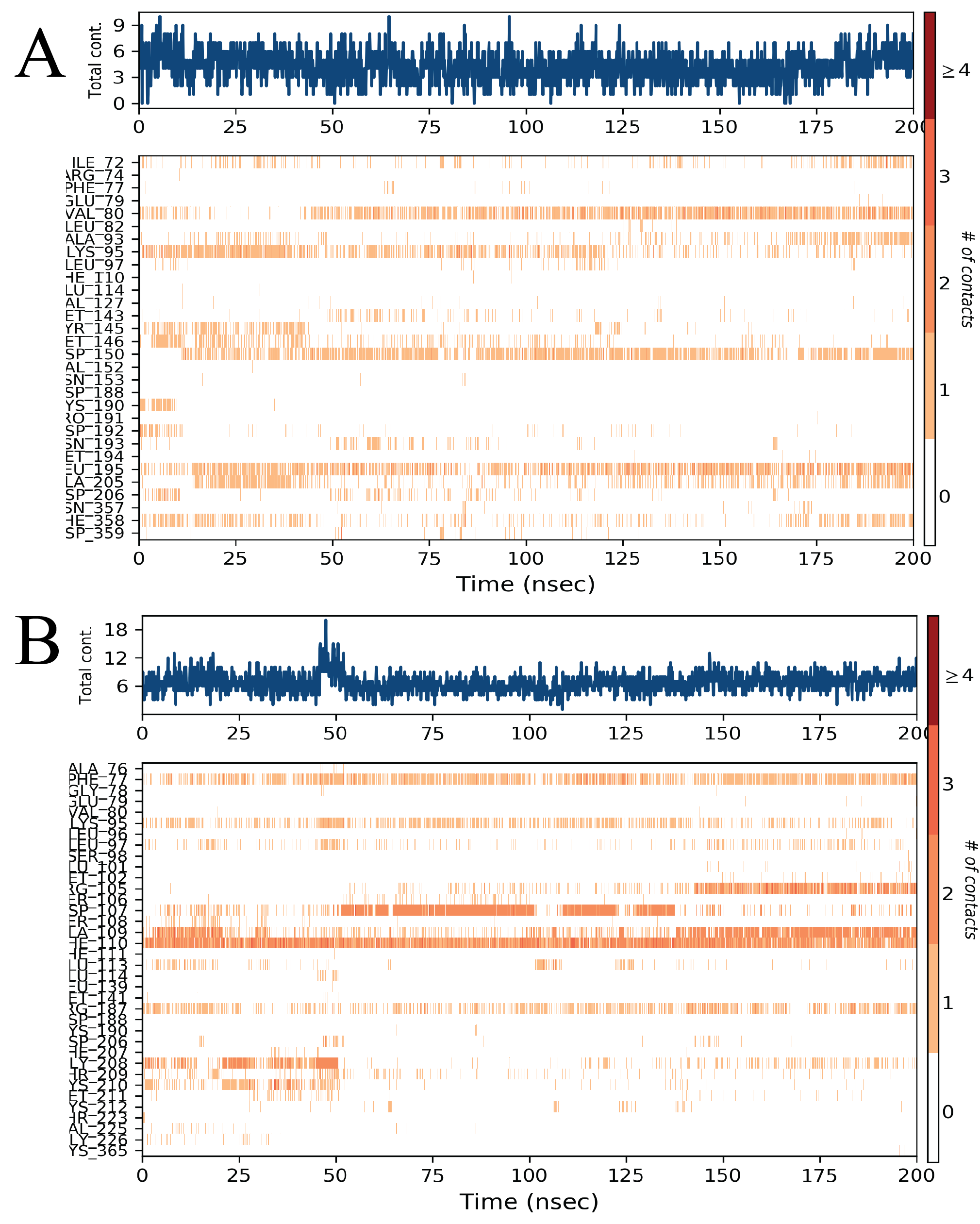


***S9 Fig. A timeline representation of interactions and different contacts such as H-bonds, Ionic, Hydrophobic, and Water bridges with A1 (A) and A2 (B).***


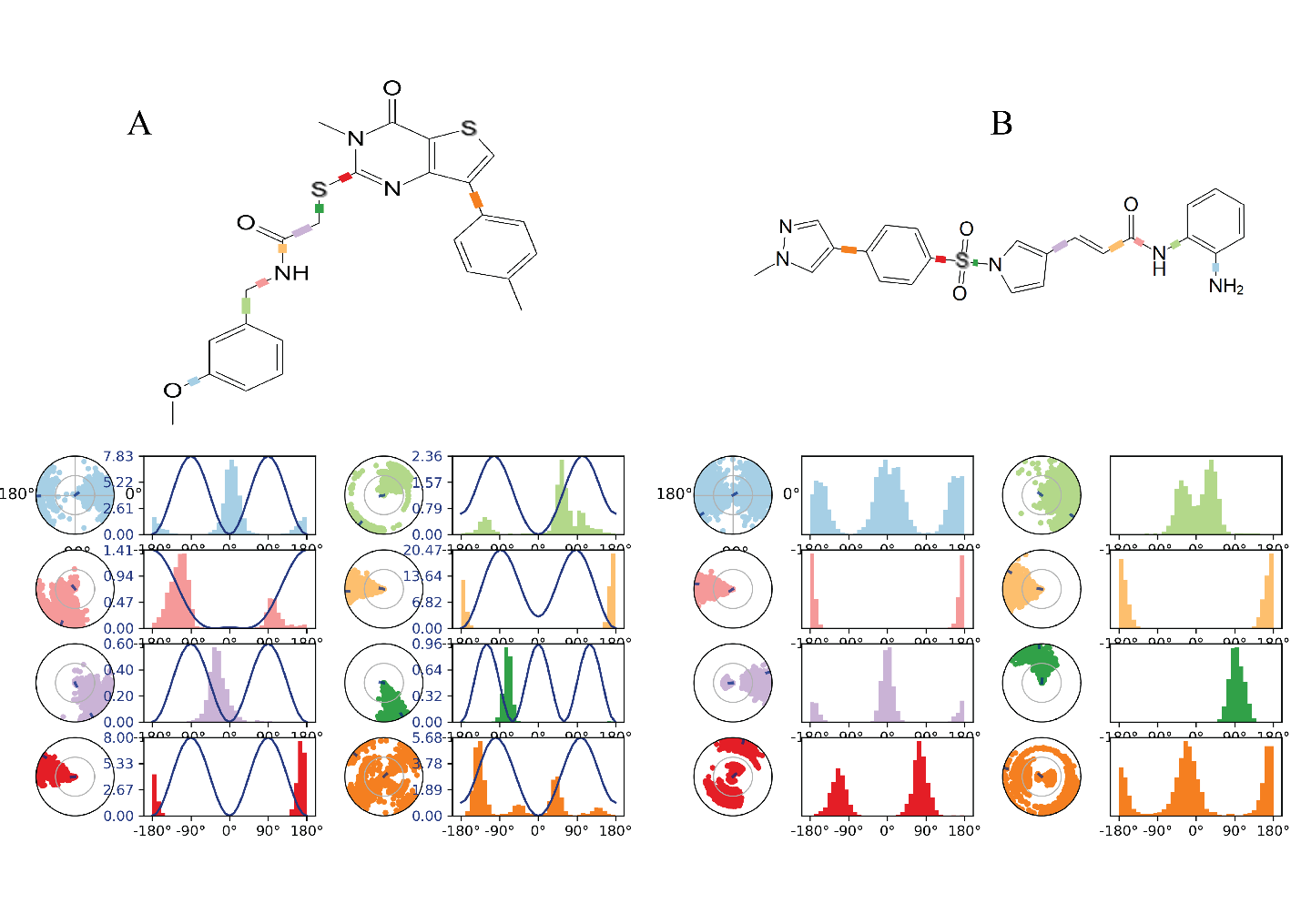


***S10 Fig. (A)Torsion plot of A1 (B) A2 represent conformational evolution of ligand’s rotatable bonds throughout the simulation. The top panel represents 2d schematic of both A1 and A2 while bottom panel shows dial plot and bar plots. The values of the potential are on the left Y-axis of the chart represented in kcal/mol.***

S1 Table. Physiochemical properties of ROCK2 receptor

| UniProt ID | O75116 |
| --- | --- |
| No of Amino Acids | 391 |
| Molecular Weight | 44972.35 |
| GRAVY | -0.316 |
| Negative Residue | 61 |
| Positive Residue | 46 |
| Theoretical PI | 5.18 |
| Aliphatic Index | 82.99 |
| Atomic Composition | C (2029), H (3103), N (529), O (589), S (20) |

***S2 Table. Binding site residues of target protein identified via CASTp and site finder tool.***

| **Protein** | **Binding Pocket Residues** |
| --- | --- |
| **Rho-associated Protein Kinase 2 (ROCK2)** | Ile72, Arg74, Gly75, Ala76, Phe77, Gly78, Glu79, Val80, Ala93, Lys95, Leu96, Asp107, Phe110, Val127, Met143, Glu144, Tyr145, Met146, Asp188, Lys190, Asp192, Asn193, Leu195, Ala205, Asp206, Gly208, Thr209, Val225, Gly226 |

***S3 Table. Active compounds along their physiochemical properties used to generate pharmacophore query.***

| Small Molecules | IC_50, and_ K_d_ | Structure | HBA | HBD | MW (KDa) | RB | LOG P | Lipinski Violation | Publications |
| --- | --- | --- | --- | --- | --- | --- | --- | --- | --- |
| J0P | 0.012 µM | 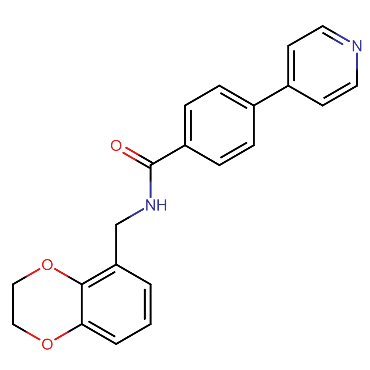 | 4 | 1 | 346 | 5 | 2.97 | NO | <https://pubs.acs.org/doi/full/10.1021/acs.jmedchem.8b01098> |
| VFA | 0.18 nM | 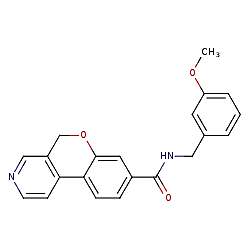 | 4 | 1 | 346.4 | 4 | 2.76 | NO | [10.1016/j.bmcl.2020.127474](http://dx.doi.org/10.1016/j.bmcl.2020.127474) |
| 3SG | 4.1 ± 1.0 | 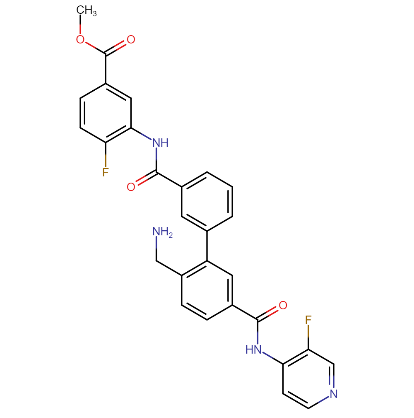 | 8 | 3 | 516 | 10 | 3.1 | 1 (M.W) | <https://pubs.acs.org/doi/full/10.1021/acs.jmedchem.5b00308> |
| VFS | 7.2 nM | 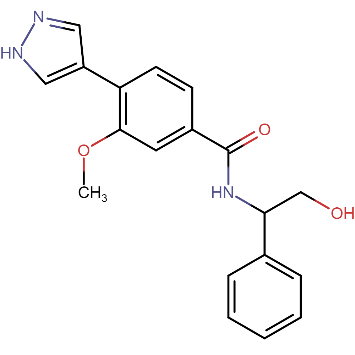 | 4 | 3 | 337 | 7 | 1.9 | NO | <https://www.sciencedirect.com/science/article/abs/pii/S0960894X20306065?via%3Dihub> |
| 1WU | 0.54 µM | 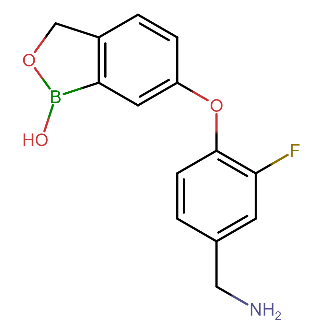 | 5 | 2 | 273 | 3 | 1.42 | NO | <https://jpet.aspetjournals.org/content/347/3/615> |
| 81G | 26 nM | 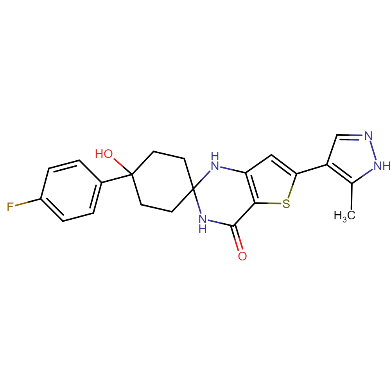 | 4 | 4 | 412 | 2 | 3.28 | NO | <https://www.sciencedirect.com/science/article/abs/pii/S0968089616313323?via%3Dihub> |
| O1V | 0.26 nM | 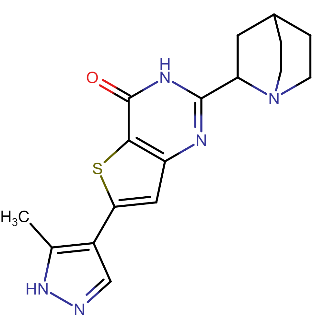 | 4 | 2 | 341 | 2 | 2 | NO | <https://pubs.acs.org/doi/10.1021/acs.jmedchem.9b01427> |
| Y27632 | 300 nM | 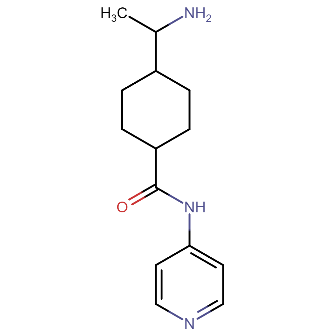 | 3 | 2 | 247 | 4 | 0.9 | NO | <https://academic.oup.com/jb/article-abstract/140/3/305/2182207?redirectedFrom=fulltext> |
| O1S | 0.26 nM | 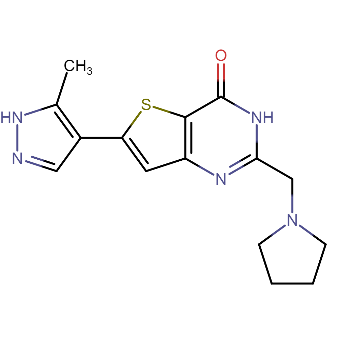 | 4 | 2 | 315.4 | 3 | 1.62 | NO | <https://pubs.acs.org/doi/10.1021/acs.jmedchem.9b01427> |
| M77 | 71 nM | 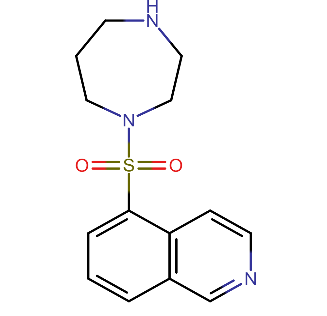 | 5 | 1 | 291 | 2 | 0.95 | NO | [10.1016/j.str.2005.11.024](http://dx.doi.org/10.1016/j.str.2005.11.024)  <https://pubmed.ncbi.nlm.nih.gov/15998434/>  <https://pubmed.ncbi.nlm.nih.gov/19061880/>  <https://pubmed.ncbi.nlm.nih.gov/16996142/> |
| Belumosudil | 60 nM | 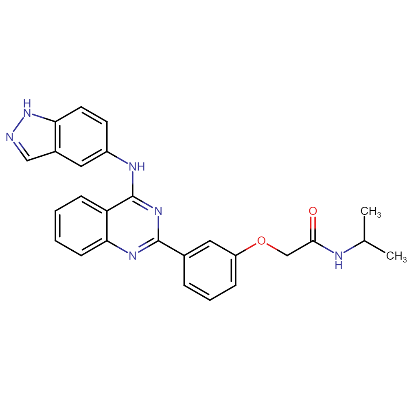 | 5 | 3 | 452 | 8 | 4.8 | NO | <https://pubmed.ncbi.nlm.nih.gov/24466563/>  <https://pubmed.ncbi.nlm.nih.gov/25385601/>  <https://pubmed.ncbi.nlm.nih.gov/26983850/> |
| Azaindole | 1.1 nM | 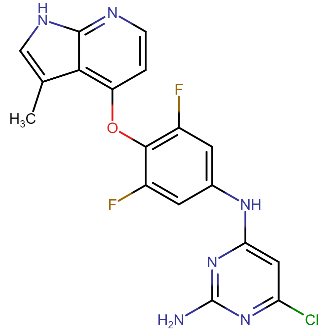 | 6 | 3 | 402 | 4 | 4.18 | NO | <https://pubmed.ncbi.nlm.nih.gov/17934515/> |
| GSK269962B | 4 nM | 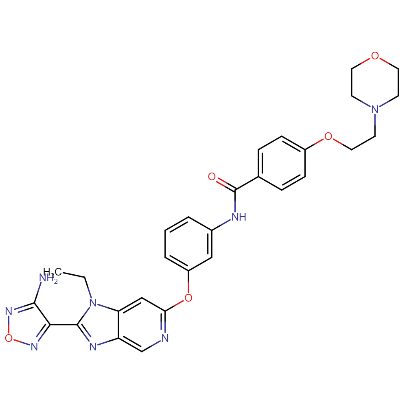 | 10 | 2 | 602 | 11 | 2.98 | Yes: 2 violations: MW>500, NoRO>10 | <https://pubmed.ncbi.nlm.nih.gov/17018693/>  <https://onlinelibrary.wiley.com/doi/abs/10.1002/nau.22978> |
| AT13148 | 4 nM | 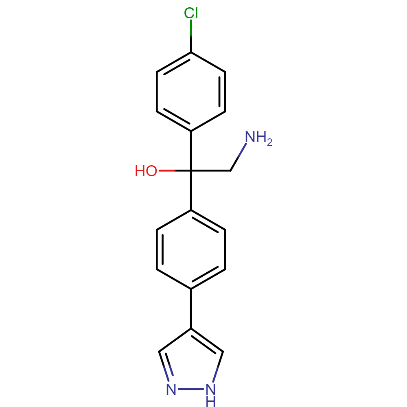 | 3 | 3 | 313 | 4 | 2.16 | NO | <https://pubmed.ncbi.nlm.nih.gov/22781553/> |
| RKI-1447 | 6.2 nM | 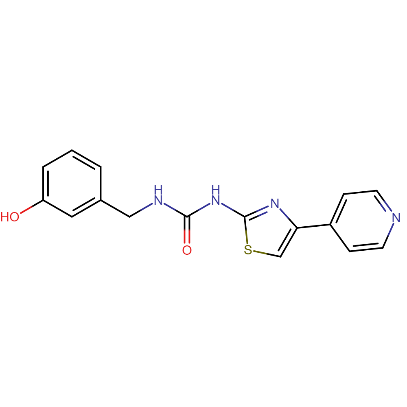 | 4 | 3 | 326 | 6 | 2.02 | NO | <https://pubmed.ncbi.nlm.nih.gov/22846914/>  <https://pubmed.ncbi.nlm.nih.gov/23275831/> |
| Ripasudil (K-115) hydrochloride dihydrate | 19 nM | 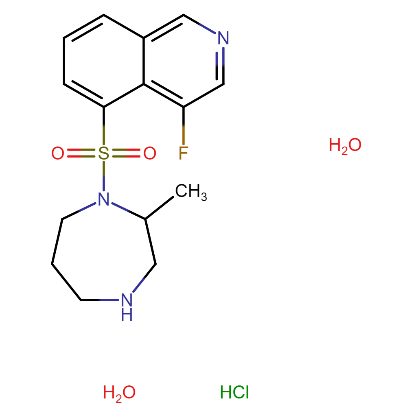 | 8 | 3 | 395 | 2 | 1.34 | NO | <https://pubmed.ncbi.nlm.nih.gov/24502505/>  <https://pubmed.ncbi.nlm.nih.gov/25277230/>  <https://pubmed.ncbi.nlm.nih.gov/26782355/> |
| GSK429286A (RHO-15) | 63 nM | 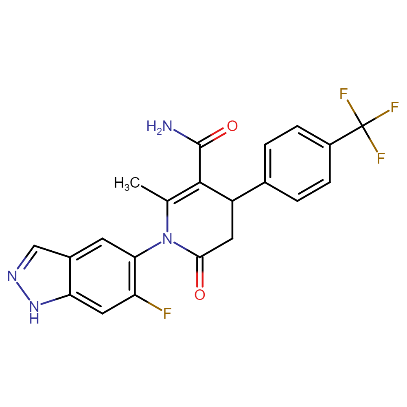 | 7 | 2 | 432 | 4 | 2.72 | NO | <https://pubmed.ncbi.nlm.nih.gov/17201405/>  <https://pubmed.ncbi.nlm.nih.gov/19740074/> |
| Hydroxyfasudil (HA-1100) | 0.72 nM | 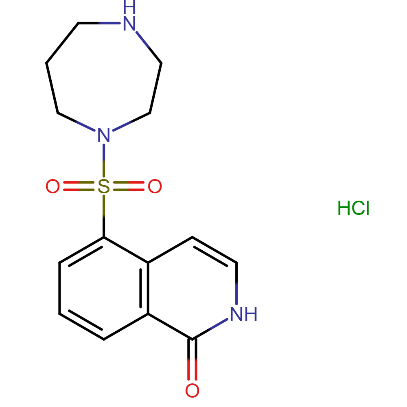 | 5 | 2 | 343 | 2 | 0.98 | NO | <https://pubmed.ncbi.nlm.nih.gov/16141422/>  <https://pubmed.ncbi.nlm.nih.gov/15708710/> |
