# Supplementary material for: Computational exploration of novel ROCK2 inhibitors for cardiovascular disease management; insights from high-throughput virtual screening, molecular docking, DFT and MD simulation
Source: PLoS One. 2023 Nov 16;18(11):e0294511. doi: 10.1371/journal.pone.0294511 (PMC10653426; doi:10.1371/journal.pone.0294511)
Supplement: Attachment — Submitted filename: Comments_plos.docx [file pone.0294511.s002.docx]

Dear Authors

This manuscript entitled as "Computational Exploration of Novel ROCK2 Inhibitors as Precision Pharmacotherapeutics for Hypertension, Mechanistic Insights and Translational Implications in Cardiovascular Disease Management" by Iqra Ali1**,** et al reported a detailed *Insilico* analysis of a wide array of molecules and identified their potential in ROCK2 inhibition. The study is framed in a nice manner and the obtained results are quite interesting to the respective field. However, I would like to suggest some refinement to the current form of manuscript, before acceptance for publication as follows.

1. Title of the manuscript is needing some sort of reframing. Without proper in-depth biological evaluation or translational studies, the present title is unsuitable or misleading. Kindly consider to revise the title according to the experiments reported in the present manuscript. For instance, according to “Precision Pharmacotherapy: Integrating Pharmacogenomics into Clinical Pharmacy Practice” by Hicks etal, “Precision pharmacotherapy encompasses the use of therapeutic drug monitoring; evaluation of liver and renal function, genomics, and environmental and lifestyle exposures; and analysis of other unique patient or disease characteristics to guide drug selection and dosing”. In this perspective, authors should justify each terminology in the title. A similar scenario can be seen in the case of “translational implications”. Kindly consider a revision in the title.
2. The language used in the manuscript needs to be improve in the manuscript. I would like to suggest a thorough revision in the overall language used. For instance, the spelling mistakes in the abstract. … Therefore, using Insilco techniques predict novel…… “I” is missing the insilico.
3. ….. 2000 best hits were selected and apply rule of 5, then docked in the binding site of ROCK2 to understand interactions of ROCK2 with hit compounds… ROCK2 is repeating in the same line.
4. In the abstract section, the only the methodology is reported. Authors should include the major results in the abstract.
5. Use of abbreviations and their expansion. Whenever using an abbreviation, kindly include the expansion on the first time use of abbreviations. For instance, …. ROCK2 target due to its substantial role in NO-CGMP and RhoA/ROCK pathway…. Authors should use this sort of methods throughout the manuscript. Though list of abbreviations are included, some places abbreviations along with the expansion is used in the manuscript. Kindly make a uniform pattern.
6. In the introduction section, first line is not clear to the reader. Kindly try to simplify the bigger statement to the simpler one.
7. … According to WHO report, there is 29% mortality rate due to CVDs… The present data is reported to the Pakistan demography or throughout the world. This is not clear from the manuscript.
8. … dysfunction of NO-cGMP pathway….. Needs to add the expansion. Kindly check this for all abbreviations.
9. It is better to add the expansions under the legend of Figure 1.
10. …. For the cure of cardiovascular diseases….. Authors should use an alternative term for cure. “Management” of the cardiovascular diseases is more suitable than the term “cure”. Kindly consider a revision.
11. Authors described some ROCK inhibitors such as fasudil, Y27632 and ripasudil in the manuscript. Are these molecules are included in the virtual screening? What is its effects in comparison to the most potential analogues in the manuscript?
12. Does any specific ROCK1 pdb structures are available? If so, what is the difference between ROCK1 v/s ROCK2.
13. …….Dual ROCK1 and ROCK2 inhibitors have been linked to problematic side effects as well as some investigators…. Kindly discuss the side effects too. Further, is it a side effects or an adverse effect? Kindly differentiate the difference between side effect v/s adverse effect.
14. Advantage/benefit over ROCK1 needs to include for a proper understanding.
15. Authors needs to discuss about the cocrystallised ligands and their interactions with the target along with their potential role. This will enable an easier comparison with the potential analogues.
16. Grid validation for the docking protocol is needs to be included in the manuscript. RMSD variation should also be included.
17. Kindly include the units of each parameters in the manuscript. For instance, in some places, the Kcal/mol is missed along with the binding score. Kindly check this for all cases.
18. Authors cross cited some tables in the manuscript. However, there are no tables in the manuscript. Kindly check this.
19. Can authors make a preliminary structural feature analysis observation made by the most active analogue and compare the same with the currently reported molecules.
