# Supplementary material for: Computational exploration of novel ROCK2 inhibitors for cardiovascular disease management; insights from high-throughput virtual screening, molecular docking, DFT and MD simulation
Source: PLoS One. 2023 Nov 16;18(11):e0294511. doi: 10.1371/journal.pone.0294511 (PMC10653426; doi:10.1371/journal.pone.0294511)
Supplement: Attachment — Submitted filename: Response Letter.docx [file pone.0294511.s004.docx]

**Response Letter**

**To**

**Emily Chenette and Ahmed A. Al-Karmalawy,**

**Editor in Chief and Academic Editor,**

**PLOS ONE**

Respected Mis,

Thank you for your kind coordination, and thanks to the reviewers for their constructive suggestions in enhancing the quality and clarity of our research. We have addressed the reviewers and corrected as per the suggestions. Please find the list below of point-by-point responses to the reviewer. Please let me know if you have any further suggestions.

**Corresponding authors**

**Dr. Abdul Rauf Siddiqi**

**Dr. Iqra Ali**

**Point-by-Point Response:**

**Reviewer: 2**

Authors have improved the manuscript in a nice manner. However, I would like to suggest some minor comments.

1. Please pay close attention to both spelling and the use of expansions. In some places, it is written as 'In-Silico,' while in others, it appears as 'insilico.' Let's ensure uniformity by carefully reviewing the entire manuscript.

**Response:** Thank you for your valuable suggestions. We appreciate your attention to detail. We carefully review the entire manuscript to ensure uniformity in both spelling and the use of expansions. We make necessary corrections for a consistent and polished final document. Your input is instrumental and improves the quality of our work.

2. A similar situation can be observed with 'NO-CGMP.' Should the 'C' be in lowercase or uppercase? Please review and make consistent adjustments throughout the document.

**Response:** Certainly, the comment's concern regarding the capitalization of 'C' in 'NO-cGMP' has been noted at some points. To maintain consistency throughout the document, the 'C' changed to lowercase, and all instances of 'NO-CGMP' adjusted to 'NO-cGMP.' Thank you for your observation.

3. Please exercise caution when using the word 'cure.' Once a person is affected by cardiovascular diseases, it typically remains with them for life. I am curious to know if there is a possibility of curing the condition with medication. To the best of my knowledge, patients need to continue taking medication throughout their lifespan for the management of condition. In this context, the use of 'cure' is inappropriate. Please correct me if I am mistaken.

**Response:** Certainly, it's essential to use precise terminology when discussing medical conditions and treatments. You're correct that the term 'cure' may not be the most appropriate when addressing cardiovascular diseases. However, we use the word 'treat' instead of cure. 'Treatment' more accurately reflects the ongoing care required to manage cardiovascular diseases, and I appreciate your input in ensuring accurate communication in this context. Thank you for the suggestion.

4. I am delighted to see that standard drugs such as fasudil, Y27632, and ripasudil have been employed in the preparation of pharmacophore modeling. However, I am curious about the effects of these molecules in the docking experiments. Including these results would facilitate a better understanding of the potential by comparing various parameters. I suggest incorporating this study into the manuscript.

**Response:** We'd like to inform you that we already compared the results of our docking experiments with standard compound 3SG (S6 Fig.) but now upon your request we dock all three enlisted standard drugs i.e., Fasudil, Y27632. However, considering the amount of data and the potential complexity, we made the decision to include the results (S5 Fig.) in the supplementary material while a bit description in the manuscript. This approach was taken to maintain the clarity and flow of the primary manuscript, while still providing interested readers with access to the additional data for a more in-depth analysis. We believe that this arrangement allows for a comprehensive examination of our findings without overcomplicating the main text.

5. The representation of 'kcal/mol' is incorrect in many parts. Please review and update accordingly.

**Response:** Thank you for pointing out the error in the representation of 'kcal/mol'. We reviewed the content and made the necessary corrections to ensure accuracy.

6. The units for RMSD values are missing in the table.

**Response:** The missing units for RMSD values in the table have been added. Thank you for bringing this to our attention.

7. Similarly, the units for distance are also missing.

**Response:** To address this concern, the distance units have been included. Thank you for bringing this to our notice, and we appreciate your comments.
